# Darcin: a male pheromone that stimulates female memory and sexual attraction to an individual male's odour

**DOI:** 10.1186/1741-7007-8-75

**Published:** 2010-06-03

**Authors:** Sarah A Roberts, Deborah M Simpson, Stuart D Armstrong, Amanda J Davidson, Duncan H Robertson, Lynn McLean, Robert J Beynon, Jane L Hurst

**Affiliations:** 1 Mammalian Behaviour & Evolution Group, University of Liverpool, Neston CH64 7TE, UK; 2 Protein Function Group, University of Liverpool, Liverpool L69 7ZJ, UK

## Abstract

**Background:**

Among invertebrates, specific pheromones elicit inherent (fixed) behavioural responses to coordinate social behaviours such as sexual recognition and attraction. By contrast, the much more complex social odours of mammals provide a broad range of information about the individual owner and stimulate individual-specific responses that are modulated by learning. How do mammals use such odours to coordinate important social interactions such as sexual attraction while allowing for individual-specific choice? We hypothesized that male mouse urine contains a specific pheromonal component that invokes inherent sexual attraction to the scent and which also stimulates female memory and conditions sexual attraction to the airborne odours of an individual scent owner associated with this pheromone.

**Results:**

Using wild-stock house mice to ensure natural responses that generalize across individual genomes, we identify a single atypical male-specific major urinary protein (MUP) of mass 18893Da that invokes a female's inherent sexual attraction to male compared to female urinary scent. Attraction to this protein pheromone, which we named darcin, was as strong as the attraction to intact male urine. Importantly, contact with darcin also stimulated a strong learned attraction to the associated airborne urinary odour of an individual male, such that, subsequently, females were attracted to the airborne scent of that specific individual but not to that of other males.

**Conclusions:**

This involatile protein is a mammalian male sex pheromone that stimulates a flexible response to individual-specific odours through associative learning and memory, allowing female sexual attraction to be inherent but selective towards particular males. This 'darcin effect' offers a new system to investigate the neural basis of individual-specific memories in the brain and give new insights into the regulation of behaviour in complex social mammals.

See associated Commentary http://www.biomedcentral.com/1741-7007/8/71

## Background

Pheromones are specific chemical signals, produced for communication between individuals of the same species, that trigger a specific natural behaviour or physiological process [1]. Signals can be single compounds or combinations of compounds in a precise ratio, detected either by smell or taste [2]. Ubiquitous among invertebrates, pheromones are used to coordinate many aspects of social behaviour, including sexual recognition and attraction to bring opposite sex conspecifics together for mating [3]. Vertebrates also make widespread use of chemical scent signals for within-species communication. However, the more complex individual-specific odours of mammals, combined with variable responses to scents that often depend on context and learning, have led many to suggest that mammalian scent signals generally do not trigger the specific inherent responses required to fulfil the classical definition of pheromones [2,4,5]. How then do mammals use scent signals to stimulate the individual-specific responses that underpin the typical flexibility of mammalian social behaviour?

Scents play an integral role in mediating reproductive interactions in many mammals. This includes not only the recognition and location of opposite sex conspecifics but also assessment of the suitability and, thus, the attractiveness of different individuals as potential mates [6,7]. In common with many species, male house mice advertise their location, successful territory ownership and dominance through scent marks continually deposited around their defended territory [8]. These urinary scents provide a broad range of individual-specific information on a male's genetic compatibility and quality that is used by females in mate selection, influencing the relative attractiveness of individual males and their scents [7,9]. Females range over several male territories under natural conditions, strongly preferring to mate with dominant territory owners, and approach selected males when ready to mate [10-12].

Sexual attraction which causes an animal to spend more time in the vicinity of chemical signals from the opposite sex, and much greater interest in opposite sex scents, is common to many animals, from *C. elegans *to elephants [13-15]. Although the specific chemical signals involved have not been identified, previous studies have revealed that adult female mice are inherently attracted to spend more time near scents from adult males than those from other females or from castrated males, regardless of any prior familiarity with males or their odours, as long as females can contact the scent [16-18]. Contact with male scents appears to be rewarding to females [19] as this induces a conditioned place preference such that females will continue to spend more time in a location where they had previously encountered male soiled substrate, even after the scent has been removed [20]. However, in addition to this inherent contact-mediated attraction, females also learn attraction to airborne volatiles emanating from male urine [16]. Although female mice are readily able to discriminate between male and female airborne odours [18,21], attraction to airborne odours is learned only on contact with the scent and is specific to the individual airborne urinary odour of the male [17]. This suggests that: (a) there may be an unidentified sexual attraction pheromone that elicits inherent female attraction to spend time near adult male mouse urine; and (b) contact with this sex pheromone may stimulate associative learning of a male's airborne urinary odour as a conditioned stimulus, so that, subsequently, females are attracted to that specific male. Here we show that a single sex-specific protein expressed in adult male mouse urine elicits the highly repeatable inherent attraction of females to spend more time near male than near female urinary scent marks. This involatile protein also stimulates female learning and subsequent selective attraction to the airborne urinary volatiles of individual males. We show that a simple sex attraction pheromone can elicit a flexible response to individual-specific odours through stimulating learning and memory in a complex social mammal, allowing female sexual attraction to be both inherent, yet selective towards particular males.

## Results

**Figure 1:**
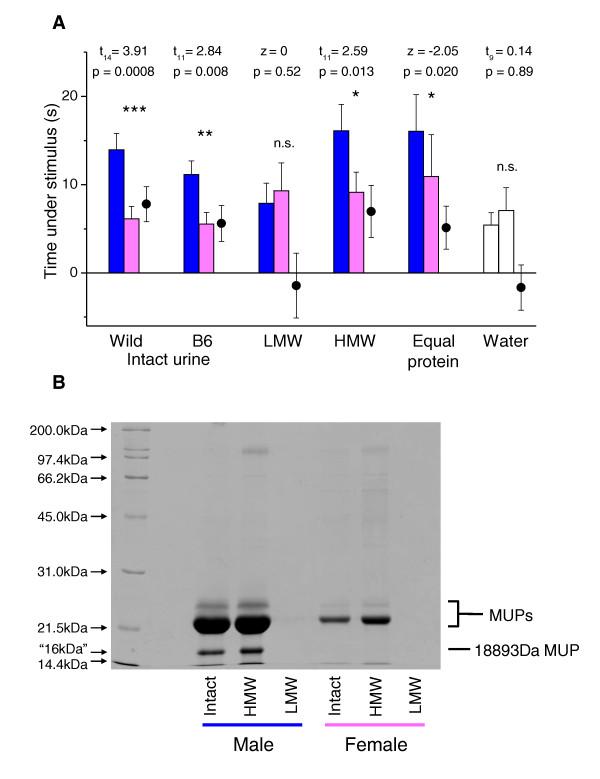
**Female sexual attraction to male urine**. (A) Total time spent under urine stimuli from males (blue bars) and from matched females (pink bars), together with the difference in time spent under male minus female stimulus (circles), plotted as means ± standard error of mean. Significant *P *values indicate greater attraction to the male stimulus (matched pair *t*-tests of log transformed data (*t*) or Wilcoxon matched pair tests (*z*) when transformed data did not approximate normality). Wild: urine from a random selection of wild males and females (*n *= 15); B6: normal intact B6 urine (*n *= 12); low molecular weight (LMW) urine fraction (< 3 kDa, *n *= 12): high molecular weight (HMW) urine fraction (≥ 3 kDa, *n *= 12); equal protein: male urine diluted to the same protein concentration as female urine (*n *= 12); water (open bars): two water stimuli (*n *= 10). The breakdown of time sniffing each stimulus and time under each stimulus not sniffing is shown in Figure S1. (B) Intact urine, HMW and LMW fractions from C57BL/6 (B6) male and female mice resolved by 15% one dimensional SDS-PAGE. Major urinary proteins (MUPs) were the only major protein bands observed, including a male-specific 18893Da MUP (darcin) which shows unusually high mobility for its size on reducing SDS-PAGE and appears as a band equivalent to 16 kDa [32].

In order to identify the molecule(s) that elicit inherent sexual attraction to male mouse urine, female house mice (*Mus musculus domesticus*) were presented with a choice between a male versus a female urine stimulus. These were streaked onto filter papers in the shape of scent marks, placed 28 cm apart on the Perspex ceiling of a clean test chamber, such that females could make nasal contact with each stimulus by stretching upright on their hind legs (full details are given in Materials and Methods); wild mice normally spend very little time in this central area of the arena, preferring to stay close to the outer walls. We used a large number of wild-derived female house mice in oestrus or proestrus as subjects throughout this study (bred in captivity to control experience). This ensures that any attraction to male scent is a general response across normal genetically-variable female mice rather than a response that is specific to a laboratory strain, and has not been influenced by many generations of artificial selection for successful breeding in the laboratory. It also avoids the problem of unnatural genetic similarity between subjects and stimulus donors that would be implicit with inbred laboratory mice [22]. All male scent donors were unrelated to the subject females, singly housed (not subordinates), in good health and their scents were not competitively countermarked [8], so should be attractive to females. In order to standardize the stimuli used in different fractionation experiments, we used pooled urine from C57BL/6 (B6) inbred mice after first confirming that female attraction to male urine from this inbred strain was as strong as that towards urine from many different wild-derived house mice (Figure 1A).

### Inherent sexual attraction through scent contact

Candidate molecules in male mouse urine that are detected on scent contact include many low molecular weight (LMW) androgen-dependent volatiles detected through V1R receptors in vomeronasal sensory neurons [23-25]. In addition, V2R vomeronasal receptors detect high molecular weight (HMW) involatile proteins and peptides including major urinary proteins (MUPs) [26,27], exocrine-gland secreting peptides (ESPs) [28] and synthetic peptides that emulate major histocompatibility complex (MHC) peptide ligands [29]. All of these molecules have been implicated in sexual signalling in the mouse, although ESPs are secreted in tears and saliva rather than in urine [30]. Separation of urine into HMW and LMW fractions by centrifugation through a 3 kDa molecular filter revealed that the LMW fraction stimulated little interest but the HMW fraction stimulated similar attraction to intact urine (Figure 1A). This was due largely to the time spent under the stimulus without sniffing (Additional File 1: Figure S1 shows the time under the stimulus broken down into sniffing and not sniffing), a more robust measure of attraction than the time spent sniffing in order to gather information from the scent [17,31]. The main components of the HMW fraction are urinary proteins (essentially all MUPs, Figure 1B) and their bound ligands (Table 1). The male stimulus contained approximately five times more urinary protein than the female stimulus (the HMW fractions of both sexes contained approximately double the concentration of urinary protein in intact urine). However, higher protein concentration *per se *was not responsible for the greater attraction to male scent: dilution of intact male urine to the same protein concentration as female urine did not eliminate the attraction response (Figure 1A).

**Table 1:** Relative concentration (counts adjusted relative to standard) of two urinary volatiles in intact B6 urine and in high molecular weight (HMW ≥ 3 kDa) and low molecular weight (LMW < 3 kDa) fractions.

	**2-*sec*-butyl 4,5 dihydrothiazole**		**3-4 dehydro-*exo*-brevicomin**	
	**Male**	**Female**	**Male**	**Female**
	
Intact urine	33	0	1.5	0.4
HMW	48	0	1.8	0.7
LMW	0.9	0	0.3	0.1
Water control	0	0	0	0

**Figure 2:**
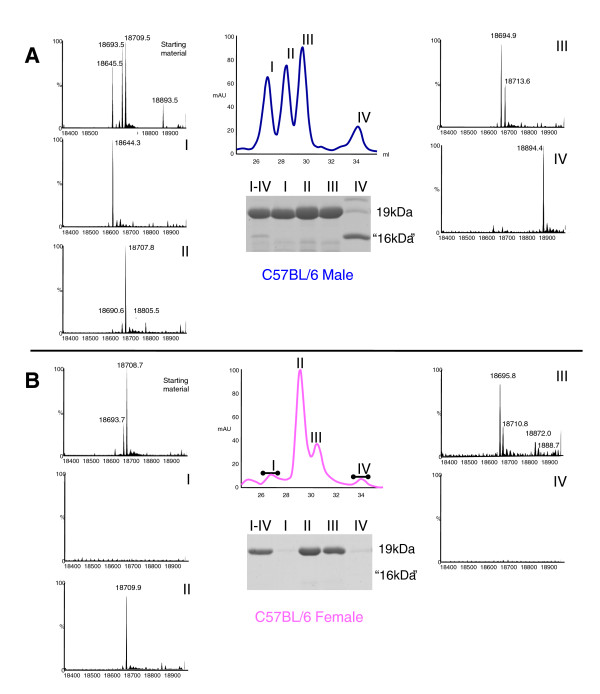
**Separation of urinary proteins in B6 mouse urine by anion exchange chromatography**. Urinary proteins from (A) adult male and (B) adult female B6 mice were separated by strong anion-exchange chromatography, monitored by ultraviolet (UV) absorbance (central trace, blue or pink line). Four specific fractions corresponding to peak UV absorbance were collected from male urine, together with the corresponding fractions from female urine (I - IV). The protein masses in each of these four fractions, along with unfractionated MUPs, were analysed by electrospray ionisation mass spectrometry (outer traces), confirming that each mass corresponded to a known MUP mass in B6 mice [28]. Male fractions I and IV each contained a single male-specific MUP with a mass that was not detectable in matching female fractions. Resolution of each fraction on SDS-PAGE confirmed the absence of MUPs in female fractions I and IV, while male fraction IV contained the male-specific high mobility 18893Da MUP, darcin.

As the main components of the HMW fraction consisted of a mixture of different MUP isoforms and their bound ligands [32,33], these were further separated by anion exchange chromatography into four specific charge group fractions (I - IV) that contained different MUPs. The same four fractions were collected from B6 female urine, although female fractions I and IV contained very low levels of protein (Figure 2, Table 2). MUPs predominated in these fractions. Intact mass analysis by mass spectrometry confirmed that male fractions I and IV contained distinct MUPs expressed predominantly or exclusively by males (masses 18645Da and 18893Da, respectively, corresponding to known *Mup *genes that show sex-specific expression in the B6 mouse strain [33]). The MUPs in fractions II and III were expressed by both sexes (Figure 2) albeit at different concentrations (Table 2).

**Table 2:** Protein concentration of each charge fraction from B6 urine and combined stimulus.

Stimulus		Protein (μg/μL)
Combined fractions I-IV	male	3.57
	female	0.47
Fraction I	male	2.96
	female	0.25
Fraction II	male	3.38
	female	1.39
Fraction III	male	3.32
	female	0.59
Fraction IV	male	0.69
	female	0.10

**Figure 3:**
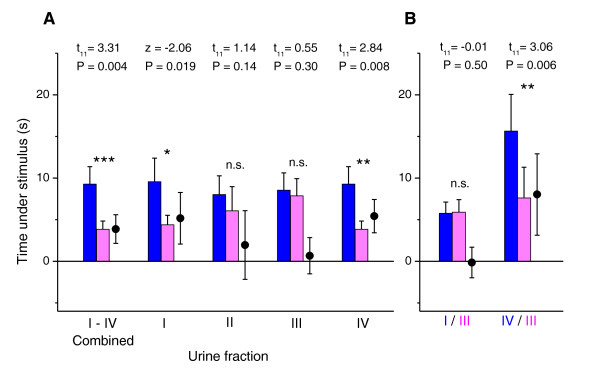
**Female sexual attraction to urine fractions separated by anion exchange chromatography containing different major urinary proteins (MUPs)**. Total time spent under urine stimuli from B6 males (blue bars) and from matched females (pink bars), together with the difference in time spent under male minus female fraction (circles), plotted as means ± standard error of mean. Significant *P *values indicate greater attraction to the male stimulus (matched pair *t*-tests of log transformed data (*t*) or Wilcoxon matched pair tests (*z*) when log transformed data did not approximate normality); n.s. = no significant attraction to male. (A) Response to urine fractions containing different MUPs (separation shown in Figure 2). I: 18645Da MUP (in male fraction only); II: 18709Da MUP (both sexes); III: 18694Da and 18713Da MUPs (both sexes); IV: 18893Da MUP (darcin, male only). The protein concentration in each male and female fraction is given in Table 2. (B) Male fractions I and IV were subsequently tested against female fraction III. For each test n = 12 except fraction I which bordered on significance and sample size was increased to *n *= 17.

A mixture of these four MUP-containing fractions (excluding all other protein-free fractions) presented at concentrations similar to that in intact urine was highly effective in eliciting much greater time near the male stimulus (Figure 3A). The difference was due to the time under the stimulus but not sniffing rather than to active sniffing behaviour (Additional File 1: Figure S2 shows the breakdown of the time sniffing and not sniffing) and was thus similar to the response to the complete HMW fraction. When each charge fraction was tested separately, fractions II and III failed to stimulate significant preference for the male fraction (Figure 3A), although the MUP concentration was much greater in the male than in the female fraction in both cases (2.4 and 5.6 times greater, respectively; Table 2). Male fractions I and IV both stimulated greater attraction than the equivalent female fractions. As this might have been due to the absence of detectable MUP in female fractions I and IV (which would not be the case for intact urine signals), we tested the attraction to each of these male fractions when paired with female fraction III (the most attractive female fraction when tested against the equivalent male fraction). Although male fraction I contained five times more protein than female fraction III, the male fraction was not more attractive (Figure 3B). By contrast, male fraction IV elicited strong attraction compared to female fraction III (Figure 3B); these male and female fractions contained approximately equal concentrations of urinary protein but very different MUP isoforms.

**Figure 4:**
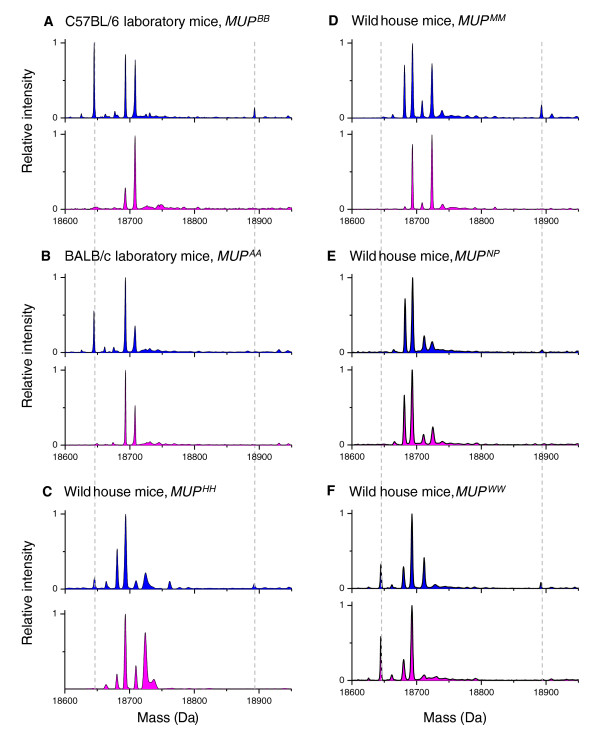
**Variation in expression of the 18645Da major urinary protein (MUP) among laboratory and wild mice with different MUP genotypes**. Electrospray ionisation mass spectra of urine samples with MUP Intensity expressed relative to the highest peak in each spectrum. Blue: male profiles; pink: female profiles; dashed lines highlight 18645Da and 18893Da. All laboratory mice examined to date exhibit strong male-biased expression of an 18645Da MUP (examples A and B). Some wild mouse MUP genotypes result in similar male-biased expression of an 18645Da MUP (C), some have no detectable expression of this mass (D and E) while some express the protein in both sexes (F). Note that the atypical 18893Da MUP (darcin) does not give a signal on electrospray ionisation mass spectrometry that reflects its abundance.

In house mice, most urinary MUP isoforms are encoded by *Mup *genes in the central region of the *Mup *cluster on chromosome 4 where there has been recent rapid expansion of gene-pseudogene pairs [33]. Variation in expression of these MUP isoforms, which differ in sequence from each other by only a few amino acids, underlies the strong individual variation in MUP profiles between wild mice. These central *Mup *genes encode the urinary MUPs expressed by both sexes as well as the 18645Da isoform in male fraction I. While expression of the 18645Da MUP is male-specific among laboratory strains [33], it is expressed only by some wild mice and male-specificity varies between wild MUP genotypes (Figure 4). By contrast, the 18893Da MUP isoform in male fraction IV is encoded by a gene in the peripheral region of the *Mup *cluster where there is considerably greater divergence between *Mup *genes [33]. This protein is variously known as the '18893Da MUP', 'Peripheral region 2 MUP 17', 'Class A MUP 24' or 'MUP20' but may be best referred to according to the Mouse Genome Informatics Database (MGI) nomeclature: (MGI: Mup20, major urinary protein 20, MGI:3651981; Ensembl reference Gene: Mup20, ENSMUSG00000078672). This MUP is consistently expressed in the urine of male but not female wild mice across a large number of genotypes ([32] and unpublished observations). It shows unusually high mobility on reducing gel electrophoresis (Figure 1B), and is responsible for binding most of the male-specific volatile 2-*sec*-butyl 4,5 dihydrothiazole (thiazole) in mouse urine [32]. In order to highlight its unusual characteristics compared to all other known MUPs, and its role in female sexual attraction (see below), we named this 18893Da MUP as darcin (after Jane Austen's hero in *Pride and Prejudice*).

**Figure 5:**
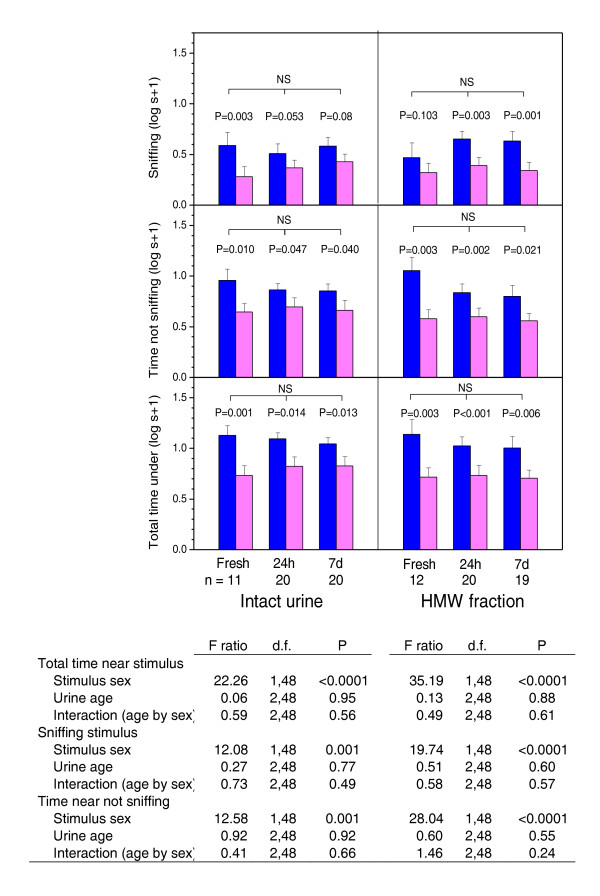
**Female sexual attraction to intact urine or to the high molecular weight fraction of urine (≥ 3 kDa, HMW) according to stimulus age**. Urine stimuli (10 μL) from male (blue bars) and female (pink bars) B6 mice were streaked onto 55 mm diameter glass microfibre filters cut in half and left to dry at room temperature for 5 min, 24 h or 7 days prior to testing. The effect of stimulus age and donor sex on response (log s + 1) to intact urine or to HMW was compared by repeated measures ANOVA with stimulus sex as a within-subjects factor and urine age as a between-subjects factor. P values on the graph show post-hoc t-tests which confirmed greater response to the male sample within each test. Log transformed data are plotted to illustrate the similarity of response between tests.

The tight binding of thiazole to darcin considerably extends the release of this volatile pheromone from scent marks over many hours; when artificially displaced from MUPs, thiazole evaporates from scent marks within minutes [8]. Thus, greater attraction to male fraction IV may be due to the binding of thiazole (and possibly other volatile ligands) to darcin. In order to explore this possibility, we compared attraction to male urine that was freshly deposited with attraction to urine deposited 24 h or 7 days prior to testing. Virtually all (> 98%) thiazole is lost 24 h after deposition under similar conditions [32]. If females are attracted by volatile components specifically bound to darcin in male fraction IV, attraction should reduce as scent marks age and volatiles are lost. However, scent age had no significant effects on the strength of attraction to the male sample whether intact urine or HMW stimuli were used in tests (Figure 5) and females continued to show significant attraction even when urine deposits were aged by 7 days.

### Attraction to recombinant darcin

**Figure 6:**
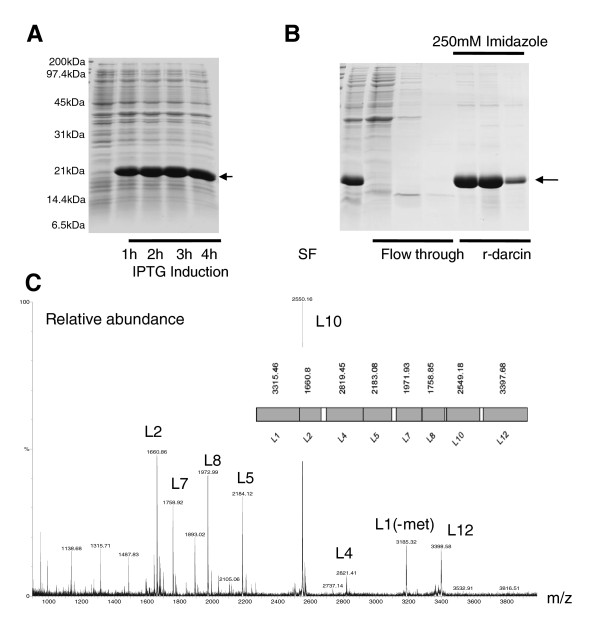
**Expression and purification of recombinant darcin (r-darcin)**. E.coli BL21(λ)DE3 cells wer e transformed with a pET28b plasmid and at hourly intervals after induction cells were removed and lysed in water. The equivalent of 0.1 A600 of lysate was loaded into each lane of a 15%(w/v) polyacrylamide gel (panel A). The cell lysate was passed through a 1.2 mm filter and applied directly to a NiNTA metal affinity column column equilibrated with 50 mM sodium phosphate, 10 mM imidazole, 0.3 M NaCl pH8.0. The column was washed in the same buffer containing 20 mM imidazole and bound r-darcin was eluted by increasing the imidazole concentration to 250 mM (panel B). The purified r-darcin was subjected to in gel digestion with endopeptidase LysC and the peptides were analysed by MALDI-ToF mass spectrometry. All LysC peptides of mass greater than 800Da were readily detected in the MALDI-ToF spectrum, confirming the expression of the correct protein (panel C).

**Figure 7:**
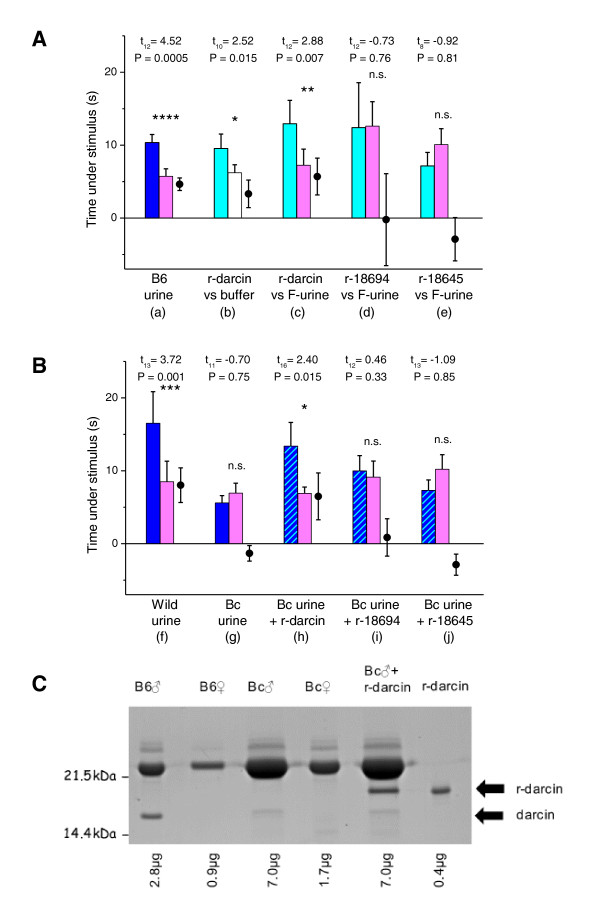
**Female sexual attraction to male urine is elicited by darcin**. Total time spent under test male stimulus (blue bars: male urine; cyan bars: recombinant major urinary protein (MUP) alone; hatched bars: male urine plus recombinant MUP) and matched control stimulus (pink bars: BALB/c female urine; open bar: buffer), together with the difference in time spent under test minus control (circles), plotted as means ± standard error of mean. Significant *P *values indicate greater attraction to the test male stimulus [matched pair t-tests of log transformed data (*t*)]. Control tests using intact urine from C57BL/6 strain [B6, A(a)] or a random selection of *n *= 14 wild males [Wild, B(f)] confirmed greater attraction to male urine. No attraction was shown towards BALB/c male urine [Bc, B(g)] which contained an extremely low level of darcin. Recombinant darcin (r-darcin, 11 μg) stimulated significant attraction when presented alone [A(b,c)] and when added to male BALB/c urine [B(h)]. There was no attraction to other recombinant MUPs (r-18694Da; r-18645Da). SDS-PAGE of urine stimuli (C), equivalent to one-thirtieth of the amount used in behavioural tests. The different mobility of r-darcin relative to native darcin is a consequence of the C-terminal His-tag used for purification (see Additional File 1: Figure S4).

Continued attraction to aged male urine does not rule out the possibility that females are sensitive to extremely low levels of thiazole that may still be bound to darcin or, perhaps, to other unidentified components in male MUP fraction IV. In order to test attraction to darcin itself, we constructed an artificial gene, codon optimized to drive high level heterologous expression of the recombinant protein in *Escherichia coli *(Figure 6 and Additional File 1: Figure S4 showing codon optimized DNA sequence and translation). The recombinant darcin (r-darcin) had atypical mobility on SDS-PAGE (Figure 7C), suggesting similar folding to the native protein. When presented alone, at a physiological level similar to that in normal wild male or B6 strain urine, r-darcin stimulated significant attraction relative to both female urine and to a buffer control [Figure 7A(b, c); Additional File 1: Figure S3 shows the breakdown of time sniffing and not sniffing]. Indeed, response levels were very similar to a B6 intact urine control test [Figure 7A(a)]. Tests of other recombinant MUPs (expressed using the same system - see Additional File 1: Figure S5 - showing codon optimized DNA sequence and translation) confirmed that females showed no attraction either towards recombinant 18694Da MUP (which does not have sex-specific expression) or towards recombinant 18645Da MUP [expressed by male but not female laboratory mice; Figure 7A(d,e)]. Note that response to the 18694Da r-MUP had abnormally high variability [Figure 7A(d)] because one female spent an unusual 85s resting under this stimulus. Excluding this trial, the mean time under the recombinant 18694Da MUP was 6.4 ± 1.3s, very similar to the time normally spent under water, buffer or female urine. This complete absence of response to other recombinant MUPs confirms that attraction to r-darcin was not due to simple unfamiliarity or to a contaminant from the expression system.

**Figure 8:**
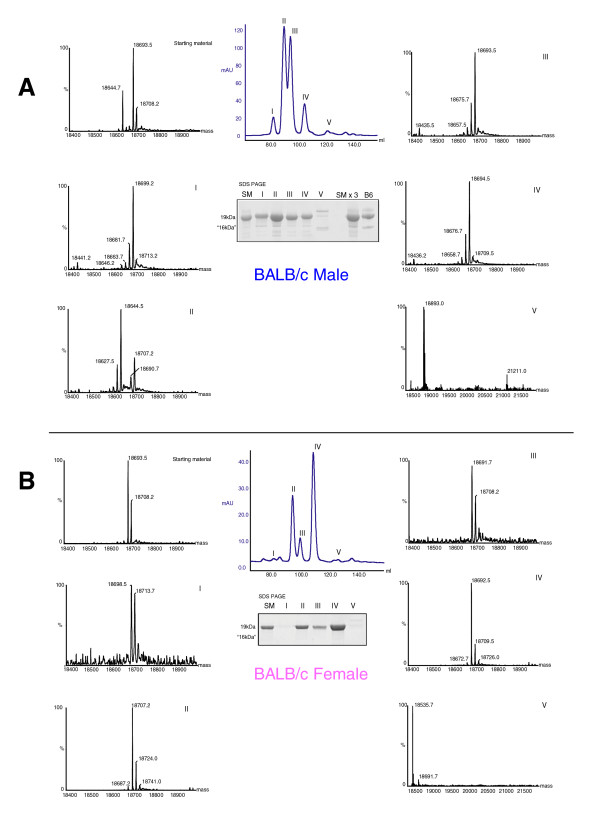
**Separation of urinary proteins in BALB/c mouse urine by anion exchange chromatography**. Urinary proteins from (A) adult male and (B) adult female BALB/c mice were separated by strong anion-exchange chromatography, monitored by ultraviolet (UV) absorbance (central trace, blue line). Five specific fractions corresponding to peak UV absorbance were collected from male urine, together with the corresponding fractions from female urine (I - V). The protein masses in each of these five fractions, along with the starting material (SM), were analysed by electrospray ionisation mass spectrometry (outer traces). Male fractions I and V contained male specific major urinary proteins (MUPs) that were not detectable in the female fractions. Resolution of each fraction on SDS-PAGE confirmed that male fraction V contained a very low abundance of the male-specific high mobility 18893Da MUP, darcin, which was not detectable in the female fraction. See Figure 1 for explanation of the molecular weight labelling on SDS-PAGE.

**Figure 9:**
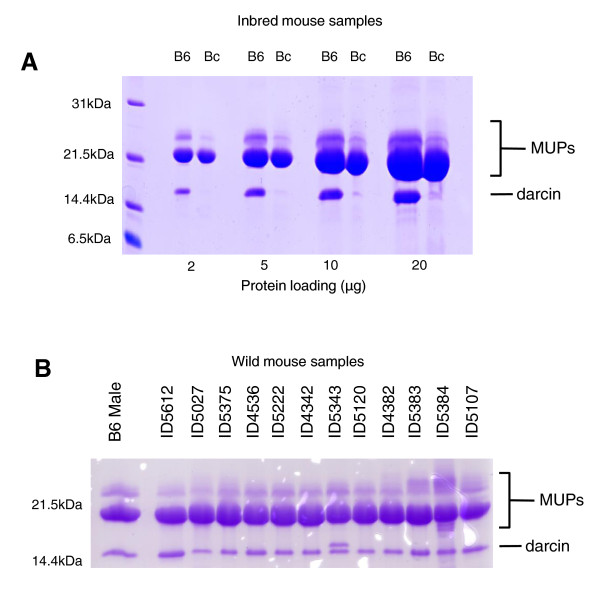
**Expression of darcin in C57BL/6, BALB/c and wild male mice**. Panel A: Urine from male adult mice of C57BL/6 (B6) or BALB/c (Bc) strains was resolved on SDS-PAGE at different loadings, from normal (2 μg of protein) to heavily overloaded (20 μg of protein) lanes. The high mobility band corresponding to darcin was evident at all loadings in C57BL/6 mice, but was barely evident at 10 μg and 20 μg loadings from BALB/c mice. Densitometric analysis of this gel indicated that darcin constituted approximately 15% of the total major urinary protein (MUP) in C57BL/6 mice, but less than 0.5% of total MUP in BALB/c mice. Panel B: Protein (5 μg) from a number of adult, male, wild-derived mice was analysed by SDS-PAGE. The darcin band, confirmed by mass spectrometry to be darcin, was evident in every animal, at levels similar to that seen in C57BL/6 mice. We do not have an explanation for the doublet in animal ID5343, but it is possible that this mouse is heterozygous for two darcin genes - the upper band has tryptic peptides that match to the darcin sequence [32].

Under normal conditions, darcin would not be encountered alone but as a constituent of urine, which also contains a large number of volatile molecules. Airborne volatiles are likely to be important for alerting attention to the presence of a scent and stimulating the close contact investigation that is necessary to detect involatile proteins [8]. As we were testing the inherent attraction that females show in response to contact with a male's scent, trials sometimes had to be excluded because females did not make contact with one or both stimulus locations during the 10 min test. When r-darcin alone was tested against female urine, there was a strong response and most females contacted the stimuli during their normal exploration of the test arena (only 2/15 trials were excluded). However, when r-darcin was tested against just a buffer control, 9/20 trials were excluded due to lack of contact. While this may have been a chance effect, the absence of any urinary volatiles in the arena may also have contributed by failing to stimulate investigation during these trials. In order to confirm that darcin elicits female attraction in the normal context of intact male urine, we also tested sexual attraction to urine from an inbred laboratory strain that expresses this MUP at only trace levels. Several of the laboratory strains derived through the Castle lineage express extremely low and sometimes undetectable levels of darcin [22], with BALB/c males expressing darcin at < 0.5% of their total MUP output (Figures 8, 9A). These classic laboratory strains have been bred in captivity over a considerable number of generations [34], divorced from the normal pressures of sexual selection and the need to attract females through scent. By contrast, adult male wild mice consistently express a high level of darcin (typically 10-20% of MUP expressed similar to B6 males, Figure 9B). As predicted, females were strongly attracted to urine from a random selection of males recently derived from the wild, but failed to spend significantly more time near male BALB/c urine than near female urine [Figure 7B(f,g)]. However, addition of r-darcin to male BALB/c urine at a normal male level stimulated a normal level of attraction [Figure 7B(h)]. They showed no such attraction when other recombinant MUPs were added to male BALB/c urine [Figure 7B(i,j)], confirming that darcin alone stimulates greater attraction to male than to female urine.

Thus, darcin acts as a sex pheromone that reliably elicits female sexual attraction to a male's urine scent, doubling the time spent near a male compared to a female urine mark. Unlike most mammalian scents that have been termed 'pheromones', we have shown that darcin meets the criteria widely accepted for the definition of the term pheromone [1,5]: it is a species-specific molecule, produced deliberately for scent signalling, and elicits a clear behavioural response that is inherent and highly consistent across the large number of genetically variable wild-derived animals used in this study. It is also equally as effective in eliciting female attraction whether encountered in urine or presented alone. We expressed darcin and other MUPs as recombinant proteins to ascertain the role of these proteins in the absence of natural ligands. We cannot exclude the possibility that r-darcin carries exactly the same thiazole in the same stereochemical configuration as the mouse, but this is extremely unlikely. First, the ligand 2-*sec*-butyl 4,5 dihydrothiazole is not a central metabolite (the biosynthesis is not known) but is unlikely to be a metabolic pathway that is essential for life. Second, a comprehensive search of the metabolic pathways of *E. coli *reveals that the only thiazole known in *E. coli *metabolism is that related to biotin synthesis, an essential metabolic function that has been lost in mammals (hence it is a vitamin). Third, as one of the other recombinant MUPs tested (18694Da MUP) also binds thiazole in mouse urine [32] it provided a good control for putative contaminant ligands from *E. coli*, and clearly stimulated no response. Thus the response appears to be to darcin alone rather than to a darcin-ligand complex.

Although the response to darcin is inherent and highly consistent, this does not mean that female sexual attraction to male mice is inflexible. Nor does this mean that other components of male urine scent have no role to play in sexual attraction. Importantly, female attraction is to the individual owner of the scent encountered, not to males in general [17,31]. As darcin is a single protein that is not polymorphic between males, it has no capacity itself for providing the individual-specific scent signatures that females need to recognize particular males, which is an essential component of selective mate choice. Instead, females learn an attraction to individual-specific airborne urinary volatiles on contact with male urine [17].

### Learned attraction to individual-specific airborne volatiles

**Figure 10:**
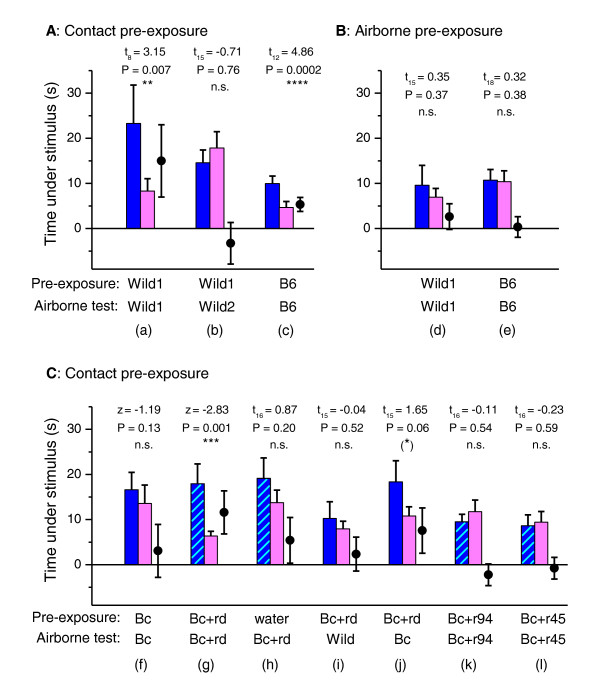
**Contact with darcin in male urine stimulates learning and subsequent attraction to airborne urinary odours specific to that individual male**. Females were pre-exposed to full contact with urine test stimuli (A, C) or to airborne odours only (B) before being tested with airborne odours from a male versus female urine stimulus that could not be contacted during the test. Displayed is the total time spent under airborne urine stimuli from males (blue bars: urine; hatched bars: urine plus recombinant major urinary protein MUP) and females (pink bars), together with the difference in time spent under male minus female stimulus (circles), plotted as means ± standard error of mean. Significant *P *values indicate greater attraction to the male airborne stimulus (matched pair *t*-tests on log transformed data (*t*), or Wilcoxon matched pair tests (*z*) when transformed data did not approximate normality). Male urine stimuli: Wild1, Wild2 = random selection of wild-derived males; B6 = C57BL/6 inbred strain; Bc = BALB/c inbred strain; Bc + rd = r-darcin added to male BALB/c urine (11 μg in 10 μL urine); Bc + r94 = recombinant 18694Da MUP added to BALB/c urine (11 μg in 10 μL urine); Bc + r45 = recombinant 18645Da MUP added to male BALB/c urine (11 μg in 10 μL urine). A standard BALB/c female stimulus was used in all tests.

To test whether it is contact with darcin in male urine that stimulates this learned attraction to individual-specific airborne volatiles, we provided females with the opportunity to learn odours by pre-exposing them to a male and female urine stimulus prior to testing. We then tested female attraction to airborne volatiles from a male versus female urine stimulus when they were unable to contact the stimuli during the test. Using urine from males that expressed normal adult levels of darcin, females learned an attraction to airborne urinary volatiles if they had prior physical contact before the test either with urine from the same individual male [using stimuli from genetically diverse wild males, Figure 10A(a)], or with urine from a genetically identical male [Figure 10A(c)]. They did not learn any attraction if they were exposed only to the airborne urinary volatiles of the male before the test without any contact with involatile cues [Figure 10B(d,e)]. We also confirmed that females learned the specific airborne odours of the individual male on contact with male urine, as females pre-exposed to contact with urine from one male showed no subsequent attraction to airborne odours that came from a different genetically distinct individual male in the test [Figure 10A(b)].

By contrast, prior contact with urine from BALB/c laboratory males, which express only trace levels of darcin (see above), failed to elicit any learned attraction to the males' airborne volatiles [Figure 10C(f)]. However, when r-darcin was added to male BALB/c urine to a level expressed by normal males, contact elicited a strong learned attraction to airborne volatiles from the stimulus [Figure 10C(g)]. This was not because r-darcin altered the airborne volatile profile such that the volatiles themselves became more attractive to females: a control test confirmed that airborne volatiles from this stimulus were not attractive if females had prior contact only with water [Figure 10C(h)]. Instead, contact with darcin stimulates females to learn the individual-specific airborne odour associated with that male's scent and subsequently show attraction only to that airborne odour; contact with r-darcin in BALB/c male urine led to no attraction to airborne odours from males with different individual airborne odour signatures [Figure 10C(i)]. The individual urinary volatile profiles of mice are complex, and are influenced by many genes including MHC [35,36], as well as by non-genetic differences such as bacterial microflora and diet [37,38]. Darcin itself may also have some influence on the pattern of airborne volatiles learned on contact: while contact with BALB/c male urine plus r-darcin resulted in strong attraction to volatiles from this same stimulus, it resulted in only weak attraction to airborne volatiles from BALB/c male urine without r-darcin [Figure 10C(g,j)]. As darcin binds and slowly releases the volatile thiazole (and possibly other volatiles) from male scent marks, it is likely to have induced a slight mismatch between volatiles in the manipulated and unmanipulated BALB/c urine. Finally, to confirm that learned attraction to airborne urinary volatiles is a specific response to darcin, other recombinant MUPs added to male BALB/c urine failed to stimulate any learned attraction to airborne volatiles from the stimulus [Figure 10C(k,l)].

## Discussion

We have shown that female sexual attraction to spend time near male urine scent is an inherent response to a single male-specific protein pheromone, darcin, detected through physical contact. This involatile pheromone also stimulates females to remember the associated airborne volatile profile and, subsequently, also show attraction to this individual-specific airborne stimulus. Contact with darcin, whether presented alone or combined with urinary volatiles, consistently doubled the time spent near a male compared to a female scent mark. Response to the airborne volatiles learned in association with darcin was even stronger, tripling the time spent near to the source of the learned individual male's scent mark relative to female airborne scent. The consistency of response is all the more remarkable in view of our deliberate use of a large random selection of genetically heterogeneous wild-stock mice to ensure that responses reflect normal functional behaviour in this species, mirroring the attraction to male urine shown by females from seminatural populations [17]. Adult males consistently express darcin at a high level in their urine and, under natural conditions, cover their territories with many hundreds of these small urine scent marks [8]. Attraction to spend time near the source of a male's scent will thus be the primary mechanism that allows females to locate a selected mate, stimulated by contact with this protein pheromone. Indeed, a wild female ready to mate will return repeatedly to a site and wait for short periods for a male to return if he is not currently present [10] (and JL Hurst's other unpublished observations).

Inherent attraction to darcin in male urine ensures that females naturally discriminate between conspecific male and female scents, without any need for social learning. However, females fail to show the same inherent attraction to male airborne urinary volatiles, despite the prevalence of many androgen-dependent volatiles in male mouse urine [39-41] that allow females to discriminate readily between male and female airborne odours [18,42]. Importantly, sexual attraction involves more than the simple recognition of opposite sex conspecifics, which could be achieved through any male-specific scent components. Females must assess an individual male's suitability as a mate, including both quality and genetic compatibility with the female's own genome [43] and then choose between those males available. The requirement for initial contact with the male-specific MUP darcin to stimulate attraction ensures that females gain full information from other involatile and volatile components of the male's scent during contact investigation, prior to showing any attraction to spend time near the male's scent. While some information may be encoded in airborne urinary odours, such as male infection status [44], we have shown that female mice depend on information encoded by other involatile MUPs in a male's urine to assess the individual attractiveness of a male according to several separate criteria: females use contact with individual-specific MUP patterns to recognize and assess the competitive ability of males from their territorial scent marks [31]; sharing of the same MUP type to avoid inbreeding with close kin [45]; and MUP homozygosity to avoid sharing nest sites with males that are likely to be inbred [46]. All of this information provides important modulation of attraction to a particular male. Other involatile protein signals detected on contact [30] may further influence a male's attractiveness, although specific behavioural responses to other involatile scent components remain to be established.

Most importantly, contact with the male-specific pheromone darcin also stimulates females to learn and become attracted to the individual-specific airborne volatiles associated with this pheromone. Thus, this pheromone signal stimulates both inherent attraction on contact and the learned individual-specific attraction that is essential for choosiness in females. Intuitively, it has been assumed that attractant pheromones in terrestrial animals will be small airborne volatiles that can be detected at a distance from the scent source [24], with involatile peptides acting as sexual attractants only in aquatic animals such as newts and other amphibia, where they can transmit through water from signaller to receiver [47,48]. However, we have uncovered a new mechanism used by terrestrial mammals such as mice, in which an involatile protein pheromone elicits the learning of associated airborne volatiles, which can then attract females to spend time near that male's scent even when they are not in direct nasal contact with the scent. The inherently attractive involatile component of male scent may itself be rewarding [19], as repeated contact with male soiled bedding in a particular location induces a conditioned preference for that location [20]; thus, females are attracted to male scent marks that they have previously contacted through the memory of their location. However, by also learning the airborne volatiles associated with the pheromone, they can then detect that male's scent wherever they encounter the same volatile profile. As many genes contribute to complex mammalian scents, the airborne volatiles learned are specific to that individual, unlike the attraction pheromone itself; this mechanism therefore allows inherent sexual attraction to be directed to particular males. We have shown previously that contact with just a single scent mark from a male induces a subsequent preference both to spend more time near to that individual scent owner and to gnaw at a barrier to get to that male [31]. At present, we do not know whether females learn all the volatiles that contribute to an individual's airborne signature or focus only on a pattern or subset of male-specific volatiles expressed by each male. Nevertheless, the high level of many androgen-dependent volatiles in male mouse urine, together with female sensitivity to these odorants, will increase the chances that a learned male's scent will be detectable, particularly from a distance. Airborne volatiles thus play an important role in selective attraction to a particular male, but this learned attraction is driven by initial contact with darcin, which elicits an inherent attraction response. The rabbit mammary pheromone (2-methylbut-2-enal), which stimulates inherent searching and grasping for a nipple in newborn rabbits, is also a potent enforcer of odorant learning [49]. A single brief pairing of any odorant with the mammary pheromone also leads to a rapid transfer of the mammary pheromone response to the odorant. Under natural conditions, pups are likely to learn the volatile odours of their mother associated with this pheromone, which then become additional direction cues for subsequent nursing.

The learning of individual odours during prior contact with scents from the opposite sex appears to be common across many mammals, resulting in memory and selective attraction to that scent owner [50-54]. However, previous studies have not suspected that this learning of individual scents may be driven by a specific pheromone, as we have shown here in mice. Notably, when inbred mice contact darcin, the airborne odours that are learned generalize to all males of that strain because they share the same genetically determined odortype. The importance of learning a particular individual male's scent, and selective attraction to this, is thus not apparent when using an inbred laboratory strain to assess the role of scents in the control and coordination of sexual behaviour. Nevertheless, individual selection is the key to female sexual behaviour outside the laboratory. However, the use of males from different laboratory strains has confirmed the importance of learning an individual male's scent in the context of pregnancy block. Exposure of recently mated laboratory females to LMW in urine constituents (< 12-14 kDa) from an unfamiliar strain male blocks pregnancy [29,55]; importantly, though, the individual scent of the familiar stud male is learned during a brief sensitive period after mating and has no such inhibitory effect [56]. Further research will be needed in order to establish whether darcin plays a role in stimulating this learning of a stud male's scent during mating or whether vagino-cervical stimulation alone provides sufficient reward to stimulate associative learning of a male's volatile urinary scent [57] regardless of the presence of darcin.

The involatile protein darcin is most likely to be detected through the accessory olfactory subsystem which detects scents that are actively pumped to the vomeronasal organ on nasal contact with the scent source [58,59]. While unique vomeronasal receptors for darcin have yet to be established, a mixture of recombinant MUPs, that included darcin, activated vomeronasal sensory neurons expressing V2R receptors [27]. Lesion of the accessory olfactory bulb (which receives input from vomeronasal neurons) is also known to abolish both the female innate preference for adult male soiled bedding and learned attraction towards airborne volatiles [60]. By contrast, the airborne individual-specific volatiles detected at a distance from the scent source are most probably detected through the main olfactory subsystem. This is activated when females explore airborne chemicals emanating from male soiled bedding or urine [41,61], with different patterns of glomerular activation in the main olfactory bulbs stimulated by airborne urinary odours from genetically distinct individuals [62]. A major future challenge will be to understand the brain pathways and neural mechanisms by which the involatile pheromone darcin stimulates the learning and memory of individual-specific airborne odours and results in attraction to this conditioned airborne stimulus.

## Conclusion

There has been much debate about whether the original concept of a 'pheromone' [1] has any useful meaning when applied to complex vertebrates such as mammals, because learning modulates most responses to odours, and the pattern of many scent components is specific to each individual [2,4,5]. Indeed, very few pheromones that trigger a defined instinctive and consistent response across genetically diverse individuals have ever been identified in mammals. Using careful molecular dissection closely coupled to assessment of a highly repeatable functional response among recently wild-derived animals, we provide evidence for the first identification of a male-specific signalling protein (darcin) that drives the inherent sexual attraction of female mice to spend time near a male's scent. Moreover, we show that contact with this protein pheromone stimulates associative learning of airborne urinary volatiles, resulting in targeted attraction to a specific individual male by choosy females. The ability of this invariant pheromone to stimulate a learned response towards individual-specific scents suggests that such pheromones may play a much more important role than previously recognized in driving flexible individual-specific social responses that are typical of mammals. Indeed, such pheromones could even underlie some complex, individual-specific responses of humans. Darcin is species-specific, like other MUPs expressed by mice, as would be expected for any pheromone that plays an important role in sexual attraction. However, MUP-like lipocalins show sex-specific expression in other rodents [63], while other scent components may be candidates for this role in more diverse species. Identifying pheromones with similar effects in other species could have important applications for manipulating mate selection in mammalian breeding programmes (among livestock, experimental animals or captive breeding for conservation). Further, the reliable response to darcin in mice could be used to investigate the neural basis of individual-specific memories in the brain that are fundamental to regulating social behaviour in mammals and other vertebrates.

## Methods

### Subjects and urine donors

The subjects were 469 captive bred adult female *Mus musculus domesticus *(F0-F2) aged 3-12 months, from a colony derived from wild ancestors captured from five different populations in the northwest of England, UK. Most females (77%) were used in only a single test over a total of 614 trials, but a small number were used in two (17%), three (4%) or four (2%) tests, each involving different scent stimuli, with 2-16 weeks between successive tests. Females were housed in 45 × 28 × 13 cm cages (MB1, North Kent Plastics, Rochester, UK) in single-sex small family groups (2-4 sisters per cage during the test period) in a different room from the urine donors. As we have previously shown that females show very similar attraction responses to urine stimuli from unfamiliar males whether completely naïve to adult male scents or after natural social and sexual experience [17], subjects were not isolated from contact with other adult male scents in the animal unit and soiled bedding from captive bred wild males was regularly added to female cages during the testing period to ensure normal oestrus cycling.

The urine donors were 17 male and 14 female C57BL/6 laboratory mice (Harlan, Loughborough, UK) aged 60 days - 9 months, 12 male and 17 female BALB/c mice (Harlan, UK) aged 4-9 months, and 33 captive bred adult male house mice derived from the same colony as the female subjects but unrelated. Males were housed singly in 43 × 11.5 × 12 cm cages (M3, North Kent Plastics, UK) to ensure that there were no subordinate males whose scent was unattractive to females. Females were housed in single sex small groups (2-4) in 45 × 28 × 13 cm cages. Urine was collected by holding the donor mouse by the scruff of the neck over a clean 1.5 ml Eppendorf tube. Urine from 5-8 individual donors of the same strain and sex was pooled for testing, using different combinations of donors to create each stimulus pool. Pooling also provided a standard female control stimulus that combined urine from donors in different stages of the oestrus cycle rather than reflecting a particular stage. Urine samples from individual captive-bred wild donor males were not pooled as each has a different individual genetic signature. Urine was collected up to 1-2 weeks prior to testing and stored at -20°C until use. A single large pool was used in each fractionation experiment to ensure that fractions were identical between behavioural replicates and biochemical analyses.

Throughout, all animals were housed on a reversed 12:12 h light cycle with lights off at 0800 h, and were maintained on Corn Cob Absorb 10/14 substrate with paper wool nest material and *ad libitum *access to water and food (Lab Diet 5002 Certified Rodent Diet, Purina Mills, MO, USA). Cardboard tubes and red plastic mouse houses (Techniplast, NJ, USA) were provided for home cage enrichment.

### Assay of attraction to male scent stimuli

Three days prior to each trial, soiled nest material and substrate from wild-derived males was introduced into the subject female's home cage to induce oestrus during the preference test [64]. Previous studies using this procedure on mice in our colony has shown that over 95% of females were likely to be in oestrus or proestrus during a test [31] and show a robust attraction to a male urine stimulus compared to an equivalent female stimulus when allowed to contact the urine stimuli [17]. All testing was carried out during the dark phase of the light cycle under dim red lighting.

Tests were conducted in a clean 45 × 28 × 13 cm arena (MB1 cage base fitted with a perforated Perspex lid [17]) to which females were familiarized for 30 min immediately before each test. In most tests, females were presented with a choice between an unfamiliar male urine stimulus and an equivalent female control stimulus that they could contact, placed within two 55 mm diameter circles that were 25 cm apart on the underside of the Perspex arena lid. Test stimuli were deliberately placed in the central zone of the test arena, where wild mice normally spend little time, so that increased time in these sites reflected attraction to the scent. In order to mimic two typical mouse urine scent marks, 10 μL of each test stimulus was streaked on to a 55 mm diameter glass microfibre filter (GMF) cut in half. These were then stuck onto the lid with Sellotape (GMF were used because the release kinetics of urinary volatiles has been characterized previously from this substrate [32,65]). The position of the male and female control stimulus was randomized but balanced to ensure that an equal number of each stimulus type was presented on each side. Trials lasted 10 min and female behaviour towards the two stimuli was recorded remotely on DVD in a neighbouring laboratory. Transcription of the DVD recordings was carried out blind to the relative position of the two stimuli during each trial which were marked as A and B.

In order to test sexual attraction to airborne urinary volatiles alone, and the influence of prior contact with male-specific urine components, females were first pre-exposed for 30 min in their home cage to a 55 mm GMF cut in half and streaked with 10 μL of each test stimulus (or water). Filter papers were held outside a mesh sphere that allowed full nasal contact with the test stimuli, or inside the mesh sphere for pre-exposure to airborne volatiles only, following the procedure in [17]. Females were then tested in the arena as described above except that fresh test stimuli were suspended 2 cm above the perforated Perspex lid using a 5 cm diameter Perspex cylinder to ensure that mice could not physically contact the test stimuli during the test.

In order to assess sexual attraction, we tested whether females spent more total time under the male rather than the female or control stimulus (nose within the 55 mm diameter circle in which a test stimulus was presented) during the 10 min trials. Time under a stimulus consisted of time spent sniffing or not sniffing the stimulus, each of which were recorded separately to provide further insight into the female's response. Time spent sniffing up at the cage lid within each 55 mm circle indicates the female's interest in gaining further information from a scent source and is part of the information gathering process, thus most sniffing occurs in the first few minutes of a test. Time under the stimulus when not directly investigating the stimulus (defined as the subject's nose being within the 55 mm diameter circle but not sniffing up at the lid) reflects non-investigatory attraction to spend time close to the scent source, and is thus likely to be a more robust measure of attraction [17,31]. In most cases, log transformation (*s*+1) of behaviour durations approximated normality (Kolmogorov-Smirnov and Shapiro-Wilks tests, *P *> 0.05) and allowed parametric analyses (matched pair *t*-tests to assess sexual attraction, parametric ANOVA to compare the log difference in response to the paired stimuli between tests). Non-parametric tests were used when log transformation did not approximate normality (Wilcoxon matched pair signed-ranks test). As it is widely established that females are more attracted to male than to female urine stimuli, and we needed to be conservative in concluding that manipulation of a urine signal eliminates this typical attraction to male urine, one-tailed statistical tests of greater attraction to male than to female or control stimuli were used throughout. In tests with scent contact, we also confirmed that the response observed after the first close sniff at a stimulus (when females may have gained involatile information from the scent) was the same as the response over the whole trial, and that time under a stimulus did not differ between the two stimuli prior to this first close contact sniff (data not presented).

### Fractionation of urine stimuli

Samples were separated into HMW and LMW components using Vivaspin 500 centrifugal concentrators (Vivascience, Sartorious Group, Aubagne, France) with a 3000Da molecular weight cut-off. Vivaspins were washed twice using ddH_2_O (500 μL added and centrifuged at 13800 rpm for 15 min). Vivaspins were emptied of water and urine (500 μL) was added and centrifuged at 13800 rpm for 15 min. The LMW component was then pipetted into a clean capped 1.5 mL Eppendorf tube, labelled and stored at -20°C until use. Urine was added to the Vivaspin to make up to 500 μL and the process repeated. The HMW and LMW components were pipette into separate capped Eppendorf tubes, labelled and stored at -20°C until use. Protein concentrations of each fraction were determined with the Coomassie plus protein assay reagent kit (Pierce, UK).

### Anion exchange chromatography

Purification of individual MUP peaks from C57BL/6 urine was achieved by high-resolution strong anion exchange chromatography on either the Duo-Flow (Biorad Laboratories, CA, USA) or the Äkta (GE Lifesciences, Bucks, UK) chromatography platforms. Both systems were fitted with a Resource Q column (GE Lifesciences, V_t _= 6 mL), equilibrated with several column volumes of 50 mM MES buffer, pH 5.0. Pooled urine was desalted on 5 mL Sephadex G-25 columns previously equilibrated with MES buffer, pH 5.0, then passed through a 0.45 μm filter. Typically 1 mL (~10 mg) of desalted protein was applied to the column. Bound protein was eluted from the column using a linear salt gradient of 0 - 1 M NaCl. Fractions (3 mL) were collected and fractions corresponding to individual MUP peaks were pooled then concentrated and desalted to deionised water (~0.2-0.3 mL final volume) using Vivaspin centrifugal concentrators (3 kD molecular weight cut off, Vivascience, Hannover, Germany). Final protein concentrations were obtained by Coomassie dye binding assay. Purity and mass was confirmed by native and SDS-PAGE as well as electrospray ionisation mass spectroscopy.

### Sodium dodecyl sulphate polyacrylamide gel electrophoresis

SDS-PAGE was performed as described by Laemmli [66]. Concentrated anion exchange fractions were mixed 1:1 with reducing sample buffer (100 mM DTT) and then heated for 5 min at 95°C. All samples were run at a constant voltage of 200 V on 15% gels. Protein bands were visualized with Coomassie brilliant blue stain.

### Electrospray ionization mass spectrometry (ESI/MS)

Concentrated anion exchange separated MUP peaks were centrifuged at 11,000 g for 10 min. All analyses were performed on an Easy-nLC nano high performance liquid chromatography system (HPLC; Proxeon Biosystems, Odense, Denmark) coupled to a QToF micro mass spectrometer (Waters, Manchester, UK), fitted with an ESI source. Samples (10 μL) were desalted and concentrated on a C4 reverse phase trap (LC packings). MUPs were eluted at a flow rate of 0.8 μL/min using repeated 0 - 100% acetonitrile gradients. Data were gathered between 800 and 1600 Th and were processed and transformed to a true mass scale using MaxENT 1 maximum entropy software (Waters Micromass, Massachusetts, USA). All data sets were processed at 0.25 Da/channel over a mass range of 18400 -19000 Da, and a peak width of 0.75 Da was used to construct the damage model. The instrument was calibrated initially using a 500 fmol/μL solution of Glu fibrinopeptide (Sigma, Munich, Germany) in 50% acetonitrile and 0.2% formic acid and calibrated during runs with a 1 pmol/μL solution of horse heart myoglobin (Sigma), 2 mM DTT in 0.2% formic acid. All water used was HPLC grade (VWR, Pennsylvania, USA)

### Gas chromatography/MS

Hexane extraction of urine and analysis of volatiles was performed according to the methodology in [32], using a Thermo Fisher Scientific PolarisQ GC/MS.

### Expression and purification of recombinant MUPs

The primary sequence of darcin was obtained from the sequence located under accession numbers NP_001012323/XP_355497 and was used to direct *de novo *gene synthesis for maximal expression in *E. coli*. The gene was codon optimised for expression in *E. coli *and cloned into pET28b via *Nco*I and *Xho*I restriction sites (Entelechon GmBH, Regensburg, Germany). The plasmid was used to transform BL21(λ)DE3 cells and darcin expressed in Luria Broth containing kanamycin (30 μg/mL). At OD_600 nm _of between 0.6-0.8, the expression of recombinant darcin (r-darcin) was induced by the addition of isopropyl β-D-1 thiogalactopyranoside to a final concentration of 1 mM. Five hours post-induction, cells were harvested by centrifugation at 2000 × g and the cell pellets stored at -20°C prior to further purification. Harvested cells were lysed using Bugbuster protein extraction reagent (Novagen, Nottingham, UK) containing Complete™ EDTA-free protease inhibitor cocktail (Roche, Burgess Hill, UK). Darcin, present in the soluble fraction of the bacterial cell lysate was purified by virtue of the hexahistidine tag on nickel affinity columns according to manufacturers protocols (Novagen). Column fractions containing r-darcin were pooled and dialysed against 50 mM phosphate, 20 mM NaCl pH7.4. This preparation was used without further processing. The purity of r-darcin was assessed by SDS-PAGE analysis and protein concentration was determined by protein assay. Similar workflows were applied to express the 18645Da MUP (accession GB/AAH91744.1) and 18694Da MUP (accession NP_001157998.1).

Recombinant darcin and other recombinant MUPs were tested alone (11 μg in 10 μL buffer) or added to stimulus urine samples (11 μg to 10 μL BALB/c male urine) in order to mimic the natural concentration of darcin observed in B6 males (approximately 10-14% of total MUP) and in wild male mice [22,32]. To ensure that both the manipulated urine and control stimulus were treated equally, an equivalent volume of 50 mM phosphate, 20 mM NaCl buffer, pH7.4 was added to the corresponding control stimulus.

## List of abbreviations

ESI: electrospray ionization; ESP: exocrine-gland secreting proteins; GMF: glass microfibre filter; HMW: high molecular weight; HPLC: high performance liquid chromatography; LMW: low molecular weight; MGI: Mouse Genome Informatics Database; MS: mass spectroscopy; MUP: major urinary protein; MHC: major histocompatibility complex; B6: C57BL/6 strain mice.

## Authors' contributions

JLH and RJB conceived the idea and gained funding for the study; SAR and JLH designed and carried out all the behavioural analyses; RJB guided all the molecular analyses. Urine fractionations and characterization were performed by SDA, AJD, DHLR and SAR; DMS and LM expressed the recombinant MUPs; JLH drafted the manuscript to which other authors contributed.

## Supplementary Material

Additional file 1 **Supplementary figures**. Figure S1: Female sexual attraction to male urine: time sniffing and not sniffing. Figure S2: Female sexual attraction to MUP urine fractions: time sniffing and not sniffing. Figure S3: Female sexual attraction to male urine is elicited by darcin: time sniffing and not sniffing. Figure S4: Expression of recombinant darcin. Figure S5: Expression of 18645Da and 18694Da recombinant MUPs. Click here for file
