# Supplementary material for: Darcin: a male pheromone that stimulates female memory and sexual attraction to an individual male's odour
Source: BMC Biol. 2010 Jun 3;8:75. doi: 10.1186/1741-7007-8-75 (PMC2890510; doi:10.1186/1741-7007-8-75)
Supplement: Additional file 1 — Supplementary figures. Figure S1: Female sexual attraction to male urine: time sniffing and not sniffing. Figure S2: Female sexual attraction to MUP urine fractions: time sniffing and not sniffing. Figure S3: Female sexual attraction to male urine is elicited by darcin: time sniffing and not sniffing. Figure S4: Expression of recombinant darcin. Figure S5: Expression of 18645Da and 18694Da recombinant MUPs. [file 1741-7007-8-75-S1.PDF]

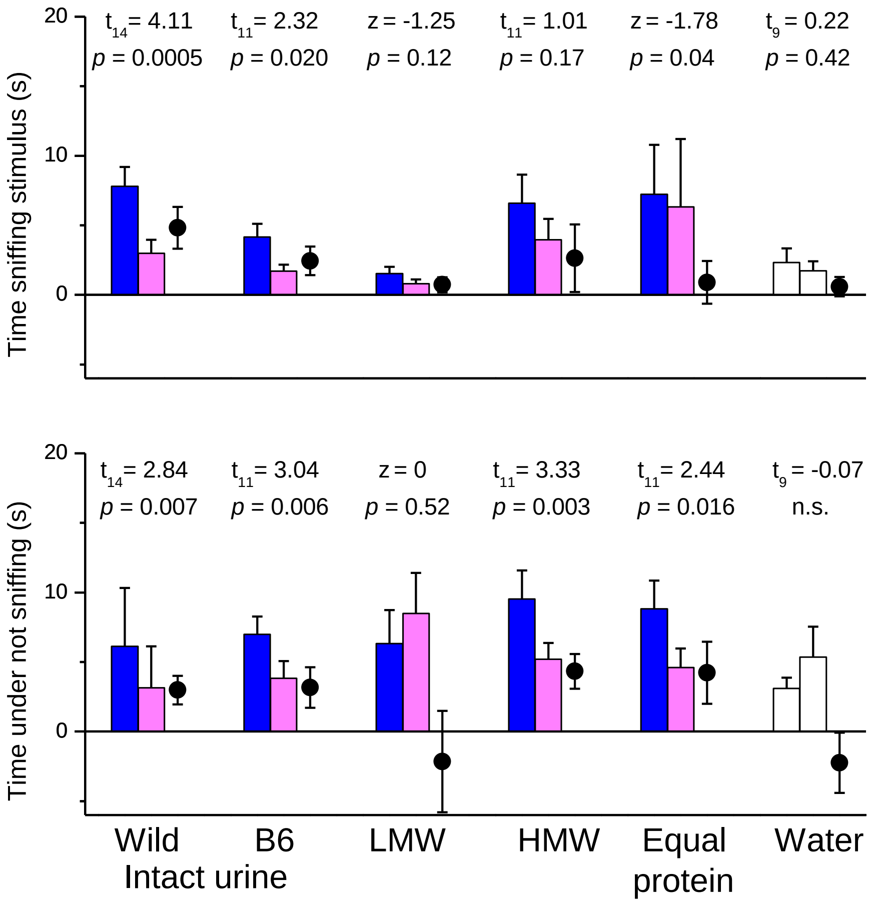

Figure S1. Female sexual attraction to male urine: time sniffing and not sniffing.
Time spent sniffing up or not sniffing when under a male (blue bars) and a matched female (pink bars) urine stimulus, together with the difference in response to male minus female stimulus (circles), plotted as means ± s.e.m. Significant *P* values indicate greater response to the male stimulus (matched pair *t*-tests of log transformed data, or Wilcoxon matched pair tests when transformed data did not approximate normality), provided as a rough indication of which fractions retained the attraction response. Wild: urine from a random selection of wild males and females (n=15); B6: normal intact B6 urine (n=12); LMW: low molecular weight urine fraction (< 3kDa, n=12); HMW: high molecular weight urine fraction (≥ 3kDa, n=12); Equal protein: male urine diluted to the same protein concentration as female urine (n=12); Water (open bars): two water stimuli (n=10). Total time under (sniffing + not sniffing) is shown in Figure 1.

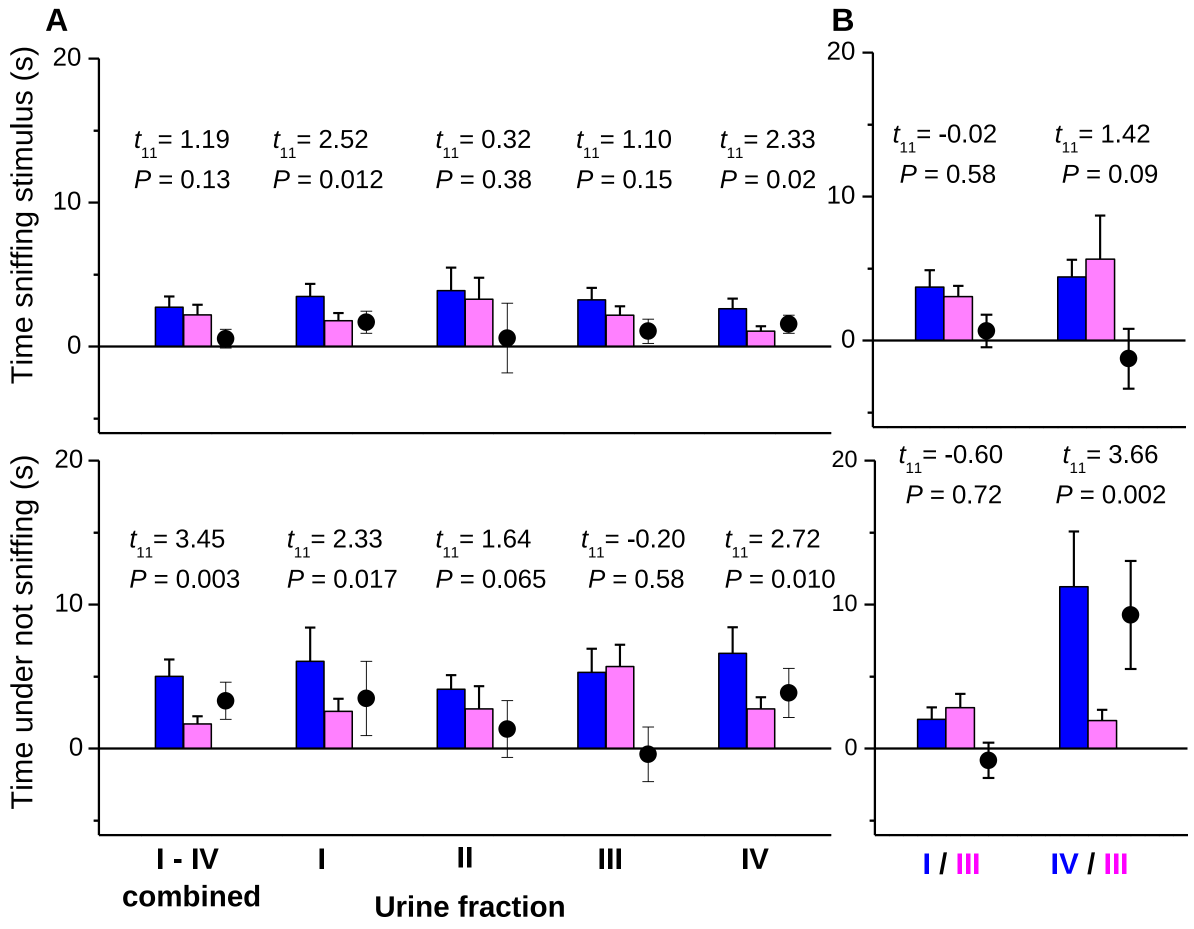

Figure S2. Female sexual attraction to MUP urine fractions: time sniffing and not sniffing.
Time spent sniffing up or not sniffing when under a male (blue bars) and a matched female (pink bars) urine stimulus, together with the difference in response to male minus female stimulus (circles), plotted as means ± s.e.m. Significant *P* values indicate greater attraction to the male stimulus (matched pair *t*-tests of log transformed data), provided as a rough indication of which fractions retained the attraction response. (A) Response to urine fractions containing different MUPs (separation shown in Figure 2). I: 18645Da MUP (in male fraction only); II: 18709Da MUP (both sexes); III: 18694Da and 18713Da MUPs (both sexes); IV: 18893Da MUP (r-darcin, male only). The protein concentration in each male and female fraction is given in Table 2. (B) Male fractions I and IV were subsequently tested against female fraction III. Total time under (sniffing + not sniffing) is shown in Figure 3.

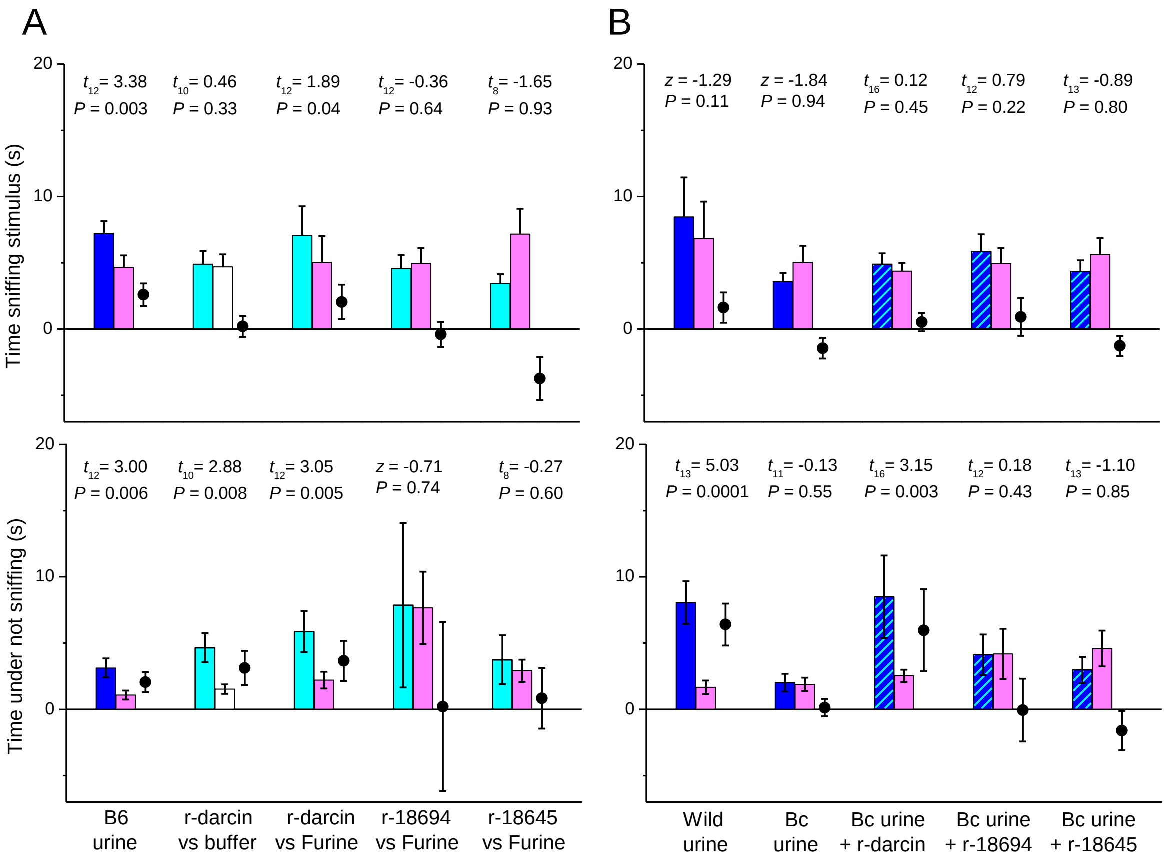

Figure S3. Female sexual attraction to male urine is elicited by darcin: time sniffing and not sniffing.

Time spent sniffing up or not sniffing when under a male stimulus (blue bars: male urine; cyan bars: recombinant MUP alone; hatched bars: male urine plus recombinant MUP) and a matched control stimulus (pink bars: BALB/c female urine with or without buffer; open bars: buffer), together with the difference in response to male minus female stimulus (circles), plotted as means ± s.e.m. Significant *P* values indicate greater attraction to the male stimulus (matched pair *t*-tests of log transformed data, or Wilcoxon matched pair tests when log transformed data did not approximate normality). Response to 11µg recombinant MUP presented alone (A) or added to 10µl BALB/c male urine (B). B6: C57BL/6 urine control; r-darcin: recombinant darcin; r-18694: recombinant 18694Da MUP; r-18645: recombinant 18645Da MUP; Furine: female urine plus buffer; Wild: wild male versus BALB/c female urine control; Bc: BALB/c urine. The total time under each stimulus is given in Figure 7.

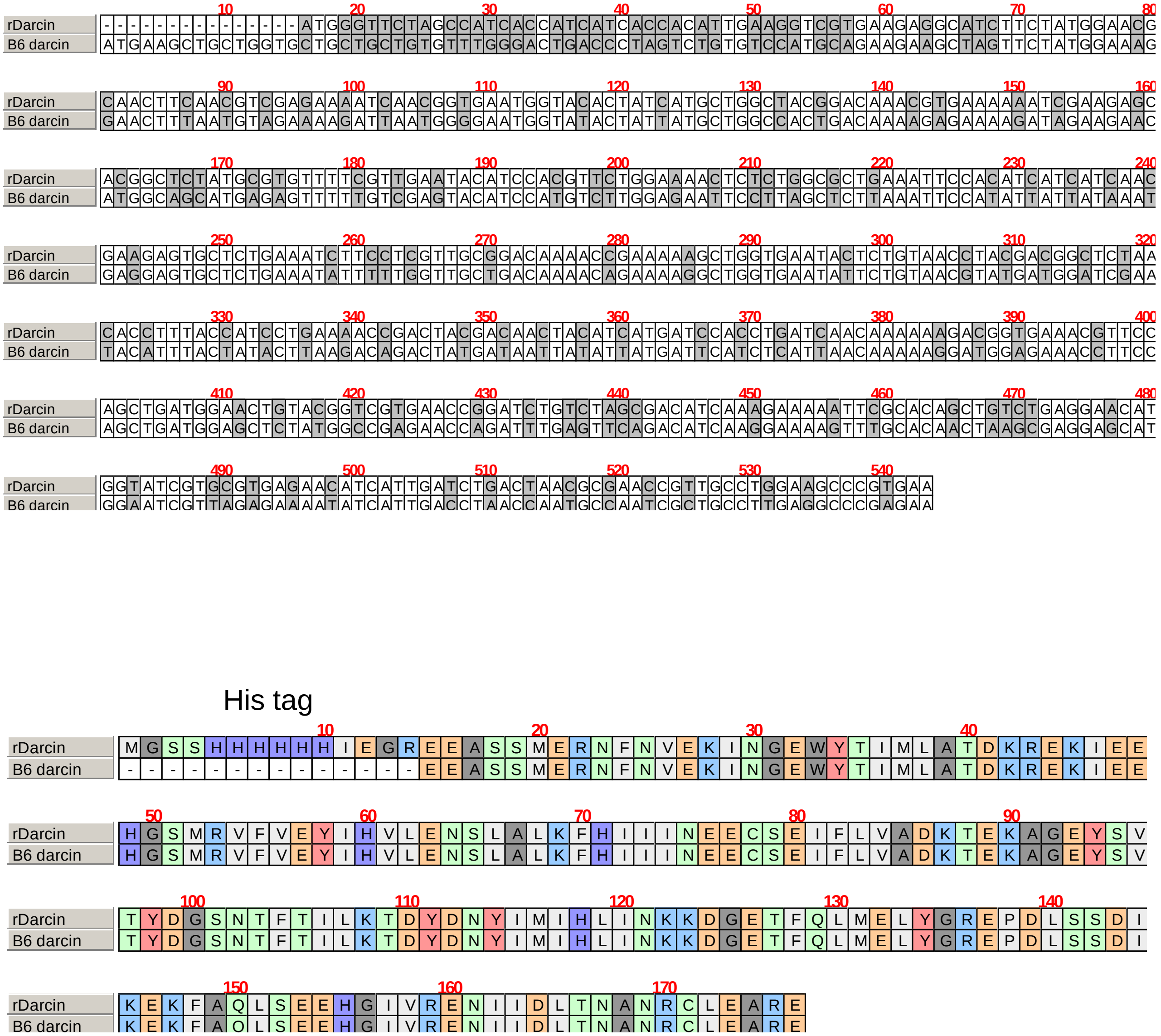

Figure S4. Expression of recombinant darcin.
The published sequence of darcin, verified by mass spectrometry was used to direct the design and synthesis of a gene, codon optimized for high level expression in *E. coli*. Top panel: The C57BL/6J cDNA sequence (B6 darcin) is aligned to the optimized DNA sequence (rDarcin). Bottom panel: translation of both sequences, emphasising the identity of the coding regions.

Translation of MUP 18645 synthetic gene, codon optimised

```
      M  G  S  S  H  H  H  H  H  H  I  E  G  R  E  E  A  S  S  T
  1 ATGGGCTCTAGCCATCACCACCATCACCATATCGAAGGTCGTGAAGAGGCATCCTCTACT
             10        20        30        40        50
      G  R  N  F  N  V  E  K  I  N  G  E  W  H  T  I  I  L  A  S
 61 GGTCGCAACTTCAACGTCGAGAAAATCAACGGCGAATGGCACACTATCATCCTGGCGAGC
             70        80        90       100       110
      D  K  R  E  K  I  E  D  N  G  N  F  R  L  F  L  E  Q  I  H
121 GATAAACGCGAAAAAATTGAAGACAACGGCAACTTCCGTCTGTTTCTGGAGCAGATTCAC
            130       140       150       160       170
      V  L  E  N  S  L  V  L  K  V  H  T  V  R  D  E  E  C  S  E
181 GTTCTGGAAAACTCCCTCGTACTGAAAGTTCACACTGTTCGTGATGAAGAGTGCTCCGAA
            190       200       210       220       230
      L  S  M  V  A  D  K  T  E  K  A  G  E  Y  S  V  T  Y  D  G
241 CTGTCTATGGTGGCTGACAAAACCGAGAAAGCTGGCGAATACTCTGTGACCTATGATGGT
            250       260       270       280       290
      F  N  T  F  T  I  P  K  T  D  Y  D  N  F  L  M  A  H  L  I
301 TTCAACACCTTCACGATCCCGAAAACCGACTACGATAACTTCCTGATGGCACACCTGATC
            310       320       330       340       350
      N  E  K  D  G  E  T  F  Q  L  M  G  L  Y  G  R  E  P  D  L
361 AACGAGAAAGACGGCGAAACCTTCCAGCTGATGGGTCTGTATGGTCGTGAACCGGATCTG
            370       380       390       400       410
      S  S  D  I  K  E  R  F  A  Q  L  C  E  K  H  G  I  L  R  E
421 AGCTCTGACATCAAAGAACGCTTTGCCCAGCTGTGTGAGAAACACGGTATCCTGCGTGAG
            430       440       450       460       470
      N  I  I  D  L  S  N  A  N  R  C  L  Q  A  R  E  *  *
481 AACATTATTGACCTGTCTAACGCGAACCGTTGCCTGCAGGCGCGTGAATAATGA
            490       500       510       520       530
```

Translation of MUP 18694 synthetic gene, codon optimised

```
      M  G  S  S  H  H  H  H  H  H  I  E  G  R  E  E  A  S  S  T
  1 ATGGGCTCTAGCCATCACCACCATCACCATATCGAAGGTCGTGAAGAGGCATCCTCTACT
             10        20        30        40        50
      G  R  N  F  N  V  E  K  I  N  G  E  W  H  T  I  I  L  A  S
 61 GGTCGCAACTTCAACGTCGAGAAAATCAACGGCGAATGGCACACTATCATCCTGGCGAGC
             70        80        90       100       110
      D  K  R  E  K  I  E  D  N  G  N  F  R  L  F  L  E  Q  I  H
121 GATAAACGCGAAAAAATTGAAGACAACGGCAACTTCCGTCTGTTTCTGGAGCAGATTCAC
            130       140       150       160       170
      V  L  E  N  S  L  V  L  K  F  H  T  V  R  D  E  E  C  S  E
181 GTTCTGGAAAACTCCCTCGTACTGAAATTCCACACTGTTCGTGATGAAGAGTGCTCCGAA
            190       200       210       220       230
      L  S  M  V  A  D  K  T  E  K  A  G  E  Y  S  V  T  Y  D  G
241 CTGTCTATGGTGGCTGACAAAACCGAGAAAGCTGGCGAATACTCTGTGACCTATGATGGT
            250       260       270       280       290
      F  N  T  F  T  I  P  K  T  D  Y  D  N  F  L  M  A  H  L  I
301 TTCAACACCTTCACGATCCCGAAAACCGACTACGATAACTTCCTGATGGCACACCTGATC
            310       320       330       340       350
      N  E  K  D  G  E  T  F  Q  L  M  G  L  Y  G  R  E  P  D  L
361 AACGAGAAAGACGGCGAAACCTTCCAGCTGATGGGTCTGTATGGTCGTGAACCGGATCTG
            370       380       390       400       410
      S  S  D  I  K  E  R  F  A  Q  L  C  E  E  H  G  I  L  R  E
421 AGCTCTGACATCAAAGAACGCTTTGCCCAGCTGTGTGAGGAACACGGTATCCTGCGTGAG
            430       440       450       460       470
      N  I  I  D  L  S  N  A  N  R  C  L  Q  A  R  E  *  *
481 AACATTATTGACCTGTCTAACGCGAACCGTTGCCTGCAGGCGCGTGAATAATGA
            490       500       510       520       530
```

Figure S5. Expression of 18645Da and 18694Da recombinant MUPs.

The published sequences of MUPs expressed by C57BL/6 mice, verified by mass spectrometry [Robertson DH, Cox KA, Gaskell SJ, Evershed RP, Beynon RJ: Molecular heterogeneity in the Major Urinary Proteins of the house mouse *Mus musculus. Biochem J* 1996, 316:265-272] was used to direct the design and synthesis of genes, codon optimized for high level expression in *E. coli*. The protein sequence (ORF) is aligned to the DNA sequence that was optimized and synthesized for maximal expression -this will differ from the published cDNA sequence.
